# Management of Bleeding in Extrahepatic Portal Venous Obstruction

**DOI:** 10.1155/2013/784842

**Published:** 2013-06-25

**Authors:** N. Chaudhary, S. Mehrotra, M. Srivastava, S. Nundy

**Affiliations:** Department of Surgical Gastroenterology and Liver Transplantation, Sir Ganga Ram Hospital, Rajinder Nagar, Room No. 2222, SSR Block, New Delhi 110060, India

## Abstract

Extrahepatic portal venous obstruction, although rare in the western world, is a common cause of major and life threatening upper gastrointestinal bleeding among the poor in developing countries. Patients have large spleens and stunted growth. The diagnosis is easily confirmed by Doppler ultrasonography. Endoscopy sclerotherapy is the best option for the control of acute variceal bleeding. For secondary prophylaxis of bleeding, the choice lies between repeated sclerotherapy and a portosystemic shunt. We believe that due consideration should be given to performing a splenectomy and a lienorenal shunt. Performed by experienced surgeons, it carries a low operative mortality of 1%, a rebleeding rate of about 10%, removes the large spleen, reverses hypersplenism, and is not followed by portosystemic encephalopathy. Most importantly, it is a onetime procedure particularly suited to those who have little access to blood transfusion and sophisticated medical facilities.

## 1. Introduction

Extrahepatic portal venous obstruction (EHPVO) is accompanied by replacement of the extrahepatic portal vein by a cavernoma with or without thrombosis of the intrahepatic portal, splenic, or superior mesenteric veins. In developing countries, EHPVO has been reported to be the most common cause of upper gastrointestinal bleeding (UGIB) in children (70% in some reports) and is also a common cause of variceal bleeding in adults [1]. In western countries, EHPVO is second only to cirrhosis as a cause of portal hypertension, but its relative incidence is much lower compared with that in the developing countries. Its aetiology is still not clear but has been attributed to umbilical sepsis after birth with thrombosis extending to the portal system via the patent umbilical vein or portal pyaemia following intra-abdominal sepsis. However, notwithstanding a lack of knowledge about its cause, most children and adults with EHPVO are generally from the so-called lower economic strata [2].

Variceal bleeding in EHPVO usually occurs in the first or second decade of life [15].

However, the outcome after a bleed is better compared to bleeding in cirrhotics (if adequate blood replacement facilities are at hand), because patients with EHPVO have normal liver function (and histology) which helps them to sustain bleeding episodes without decompensation [16]. However mortality rates of between 5 and 30% have been reported for a single bleeding episode because of the large volumes of blood lost in patients who do not have access to sophisticated medical facilities including blood transfusion [17].

Till the middle of the 20th century, surgery was the only treatment available for these patients. However, with the advent of endoscopic therapy, this soon became the predominant treatment modality for the control of acute bleeding and also an important method for the prevention of a repeated bleeding episode. The main disadvantages of endotherapy are that it requires multiple sessions and a long-term followup with a recurrence rate of up to 40% in some studies [18]. Because the prevalence of EHPVO is the highest in developing countries and the condition affects mainly the poor [2, 19], most of whom do not have access to blood transfusion facilities and are not treatment compliant, the benefits of using a less invasive procedure like endoscopic therapy must be weighed against surgery which, in the best centres carries an operative mortality of 1%, is a onetime treatment, is not associated with encephalopathy and followed by rebleeding rates of less than 10%. Moreover, operations like a splenectomy and proximal lienorenal shunt eliminate a large painful spleen and hypersplenism and restore a normal growth pattern in children. Thus, any new treatment for EHPVO must be compared with the results of shunt surgery which have stood the test of time especially if it has been performed by an experienced surgeon.

## 2. Diagnosis

A history of major upper gastrointestinal bleeding in a child who has oesophagogastric varices in endoscopy and normal liver function tests should raise the suspicion of extrahepatic portal venous obstruction. Although a similar picture can also be present in well compensated Child A, cirrhosis EHPVO is much more common in children especially in the developing world. Investigations to confirm the diagnosis are usually simple and readily available at most centres. 

Massive splenomegaly is present in 90% of patients, and a complete blood count may reveal a low haemoglobin level and decreased total leukocyte and platelet counts due to hypersplenism in 50%. Liver function tests are nearly always normal, unlike in cirrhotics, but in the long-term the prothrombin time and albumin levels may be deranged due to the prolonged decreased portal blood flow and hence decreased synthetic function [20].

**Figure 1:**
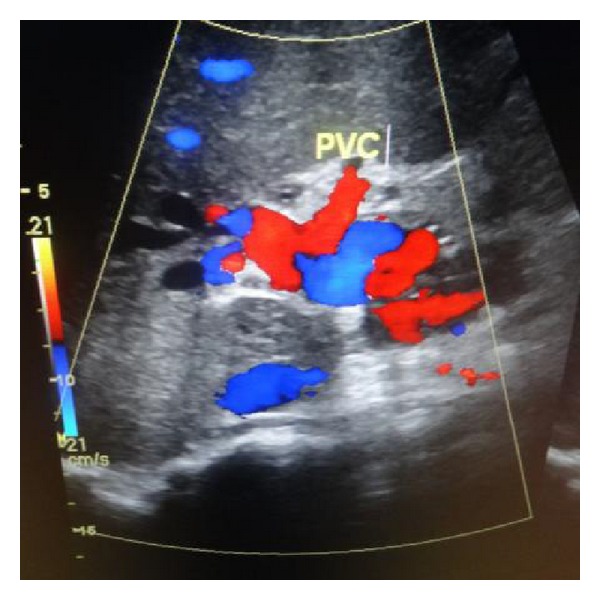
USG Doppler showing cavernous transformation of portal vein. PVC—portal vein cavernoma.

Ultrasonic Doppler (USG Doppler) examination of the upper abdomen should be the first radiological investigation performed to confirm the diagnosis as it has a sensitivity of 70–90% and a specificity of 99% in diagnosing EHPVO [21]. The characteristic findings are the replacement of portal vein by multiple tortuous vessels, also known as cavernous transformation, with hepatopetal blood flow in the collaterals (Figure 1). The liver echotexture is usually normal.

Upper gastrointestinal endoscopy (UGIE) is done to confirm the presence of varices and to grade their size. It can also identify gastric varices, portal hypertensive gastropathy, and duodenal varices.

Splenoportovenography by splenic puncture or selective coeliac/superior mesenteric angiography provides excellent imaging of the portal venous system but is invasive and has largely been replaced with computed tomography and magnetic resonance angiography. 

CT arterial portography is highly accurate, not operator dependent, and useful in circumstances when bowel gas obscures the findings on ultrasound examination. 

**Figure 2:**
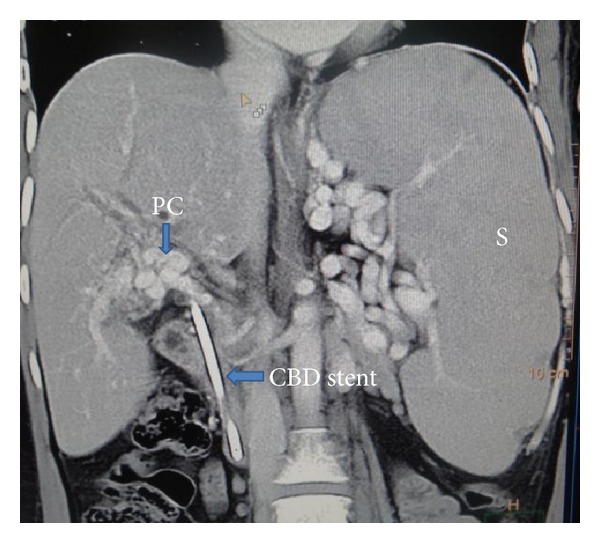
CT arterial portogram showing portal vein replaced by multiple collaterals, that is, cavernomatous transformation of the portal vein also known as a Portal Cavernoma (PC), grossly enlarged spleen (S), and CBD stent in situ for portal biliopathy in a patient with EHPVO.

However, its high cost, exposure to radiation, and the systemic toxicity of the contrast agents used are its main disadvantages (Figure 2).

MR angiography is noninvasive and has a diagnostic accuracy that is similar to CT scans. However, it has limited availability and also has a high cost. With newer MR techniques, a clear portal venogram can be obtained and the direction and velocity of blood flow in the portal system can be determined. 

Liver biopsy is usually not required for diagnosing EHPVO. It should be done only if a patient presents with abnormal LFTs or has hepatic dysfunction [20] due possibly to coincident hepatitis following repeated blood transfusions for previous bleeding episodes.

## 3. Tests to Establish the Aetiopathogenesis of EHPVO 

In spite of many years of familiarity with the disease, the causal factors have not been established. Umbilical sepsis at birth, portal pyaemia, and thrombosis, and so forth are rare clinical features. Larroche in his landmark paper in 1970 described portal vein thrombosis occurring after umbilical vein catheterization, but most of these resolved within short period of time and did not progress to EHPVO [22]. However, there seems to be a strong association with levels of hygiene and poverty as the disease seems to affect the socially disadvantaged and has all but disappeared in most western countries and Japan [19] where it was more common in the late nineteenth century. There have been occasional reports of tests for dysmorphic megakaryocytes and endogenous erythroid colony assessment as being sensitive in distinguishing these patients from a normal population [16], but these are not readily available and therefore not commonly used. Tests for venous thromboembolism such as the presence of factor V Leiden, protein C, S and antithrombin III levels, and prothrombin gene mutation may also be positive in certain adult patients [23, 24] but have not had a high yield in the Indian scenario [25]. 

## 4. Treatment 

Variceal bleeding being the most common and most serious presentation of EHPVO [17], it remains the most common indication for treatment which should be tailored depending on the patient's social circumstances and his or her access to medical facilities. Before the 1980s, conservative medical management was recommended [26–29] which included bed rest and blood transfusions as it was thought that these children would eventually “grow out” of their disease possibly by forming more collaterals to decompress the raised portal venous pressure [30]. However, when it was realized that a single episode of variceal bleeding could carry a mortality rate of up to 30% in western countries [17], most physicians now follow a more active approach. In India, Basu followed 25 patients with EHPVO for five years who had not had any intervention and reported that all had died [31]. The main treatment options available today, as with other forms of variceal bleeding, are pharmacotherapy with beta blockers, endoscopy, and surgery.

### 4.1. Pharmacotherapy

Prevention of a first episode of variceal bleeding with drugs has been well studied in adults with cirrhosis. In children with Child A cirrhosis and portal hypertension, Ozsoylu et al. [32] found propranolol to be effective. However this has not been studied in those with EHPVO. One of the most probable reasons may be that these patients usually present with bleeding as their first symptom (rather than splenomegaly), and hence primary prevention is difficult to study. Further studies are required to assess the role of drugs for primary prevention of variceal bleeding in EHPVO.

Secondary prophylaxis with beta blockers has been shown to be effective in reducing presinusoidal portal hypertension in animals and humans, but their efficacy in preventing variceal bleeding in EHPVO has not been proved [33].

### 4.2. Endoscopic Therapy

#### 4.2.1. In Acute Bleeding

Excellent results have been achieved using endoscopic therapy in the control of acute bleeding, and this has now become the therapeutic modality of choice [1] in this situation with injection sclerotherapy and elastic band ligation being effective in 90% of cases. In those few patients who continue to bleed or are bleeding so massively that endoscopic control is not a possible surgical treatment in the form of portosystemic shunts or oesophagogastric devascularisation, it has been shown to be effective [34–36] but carries mortality rates ranging from 10–30% [37]. 

#### 4.2.2. In Primary Prophylaxis

Endoscopy has also been used for primary prevention of a bleeding episode. Gonçalves et al. in a prospective randomized study including both cirrhotic and noncirrhotic children with portal hypertension found that 42% of patients with oesophageal varices who had no intervention presented with bleeding as compared to 24% patients in whom sclerotherapy was used for primary prevention [38]. However, there was no difference in survival and patients with sclerotherapy had more bleeding episodes from ectopic sites after the procedure. But since few EHPVO patients in the developing world present before a bleeding episode, endoscopic therapy for primary prophylaxis has not become an established mode of treatment.

#### 4.2.3. In Secondary Prophylaxis

 Complete eradication of varices with endoscopic sclerotherapy (EST) and variceal band ligation (EVL) occurs in 80–90% of patients with EHPVO. At present, endoscopic variceal eradication therapy is mainly indicated if

- it is used as a primary treatment modality,
- the vessels are too small for anastomosis,
- extensive thrombosis of the portal venous system which means there are no veins available for shunting, and
- in those patients who cannot tolerate the surgical procedure due to underlying comorbidities.

However it must be remembered that endotherapy does not relieve the underlying portal hypertension and may result in an increased incidence of gastric and ectopic varices [39].

EST is associated with higher rates of ulcer and stricture formation than EVL which has the additional advantages of eradicating varices in fewer sessions. A randomized study by Zargar et al. has shown superiority of EVL over EST [40], but others have found that EVL alone may be associated with increased risk of recurrence of varices (40% in one study) [41]. Poddar et al. in 2005 showed that EVL + EST compared to EST alone was a better method in treatment of oesophageal varices in children with EHPVO because of fewer treatment sessions and fewer complications [42]. They achieved a 100% eradication with EVL + EST with no postprocedure esophageal stricture. Variceal recurrence rates were low in both the groups over a followup of 27 months (6.6% in EVL + EST group as compared to 10% in the EST group). Similar results have been shown by the same author in another study [43]. Further studies are, however, needed to establish the superior efficacy of EVL + EST over EST or EVL alone. However, EST is still favoured over EVL in many places due to its low cost. 

*Long-Term Follow-up Results with Endoscopic Therapy (Table 1).* Zargar et al. have shown 88% success with injection sclerotherapy in a followup of 15 yrs [6]. Most of their patients had recurrent bleeding in the first 4 yrs after variceal eradication which was also managed by endoscopic therapy, and therefore the authors recommended annual endoscopy for the first 4 yrs after variceal eradication. In contrast, a similar study from King's College Hospital in London, UK, had previously shown that after a mean followup of 8.7 yrs, recurrent bleeding occurred in 31% [5].

### 4.3. Surgical Management

Surgery is the definitive treatment modality for EHPVO. We believe that it should be recommended as the treatment of choice for secondary prophylaxis in developing countries where the disease is more common and the accessibility to health care resources is poor. 

Surgical intervention in variceal bleeding in EHPVO is indicated if there is

- failure of endoscopic management in acute variceal bleeding,
- bleeding not amenable to endoscopic treatment such as portal hypertensive gastropathy and ectopic varices,
- as a onetime treatment for secondary prophylaxis in those who have difficult access to specialized centres
- for associated complications like portal biliopathy, growth retardation, hypersplenism, and massive splenomegaly leading to poor quality of life.

The surgical options include portosystemic shunts (PSS) and ablative procedures.

#### 4.3.1. Portosystemic Shunts

These procedures divert blood flow from the high pressure portal circulation to low pressure systemic circulation by creation of an anastomosis between a tributary of the portal vein (splenic, superior mesenteric, and left gastric, left gastroepiploic) and a systemic vein (renal, inferior vena cava, and adrenal). The shunts may be selective (i.e., only decompressing the varices) or nonselective (decompressing the entire portal venous system). The main requirement for shunt procedure is the presence of a vessel free of thrombus and of sufficient size. For shunt to be effective and remain patent, it should be at least 10 mm in diameter although Bismuth et al. [44] and Prasad et al. [9] have had patency rates ranging from 84 to 96% after anastomosing veins of down to 4 mm in diameter. 

(A) *Nonselective Shunts*. These are the most commonly performed procedures and include proximal splenorenal (PSRS), mesocaval, and portacaval shunts. The initial results with nonselective shunts were not promising since shunt thrombosis and rebleed and encephalopathy occurred in a large proportion of patients [29, 45]. But recent series have shown rebleed rates of 2–11%, a mortality rate of <2%, and no postshunt encephalopathy [9, 10]. Orloff et al. in 1994 found similar results with proximal side-side splenorenal shunts with or without splenectomy and end-side cavomesenteric shunts. They showed survival rates of >96% after 10 years and shunt thrombosis rates of <2% over a 15 yr followup [10]. 

A PSRS is the most commonly performed shunt along with a splenectomy. It is advantageous in that, along with diversion of blood flow to decrease portal pressure and control bleeding, it also relieves the patient from symptomatic enlarged spleen and the effects of hypersplenism. PSRS has shown good long-term results. In a study by Prasad et al., the 15 yr survival was 95% in 160 patients of EHPVO treated with PSRS with a rebleeding rate of 11% [9]. The long-term outcomes with shunts are shown below (Table 2). 

The rebleeding rates after shunts are also lower than that after endoscopic therapy. In a recent prospective randomized study by Wani et al. in which the authors compared endoscopic sclerotherapy and shunt surgery revealed that rebleeding rates were significantly lower in the shunt surgery group (3.3% versus 22.6%) [46]. Treatment failure rates were also much less in the surgery group (6.7% versus 19.4%). Shunt procedures may also result in an improved quality of life (QOL). A study by Krishna et al. evaluating QOL after endoscopic or surgical treatment of EHPVO showed that endoscopic variceal eradication had no significant effect on QOL, but the postsurgery group had improvement in physical, psychosocial, and total QOL scores. However, the differences were not statistically significant [47].

The risks of overwhelming postsplenectomy infection have been described, but many studies including those from India and Mexico have shown low rates of postsplenectomy sepsis [9, 48, 49]. Other disadvantage is rebleeding due to shunt thrombosis. Rates of shunt thrombosis vary from 4–16% [8–11], and rebleeding in these patients is usually easily controlled by endoscopic therapy.

Surgical shunts in cirrhotics have been shown to be associated with neurological disturbances. In EHPVO, however this complication is rare, but there have been occasional reports of abnormal findings on electroencephalography, late CNS side effects, and emotional disorders even after 20 yrs [26]. Minimum hepatic encephalopathy (MHE) rates were higher in surgically shunted patients as compared to nonshunted children (but the difference was not significant) [50]. However, most studies have shown no postshunt encephalopathy [8–11].

Shunt procedures, however, are not popular in the emergency setting because they are (i) time consuming, (ii) need technical expertise and associated with (iii) high rates of shunt thrombosis, and encephalopathy. However, the rate of shunt thrombosis depends on the experience of surgeon. A study by Orloff et al. showed shunt thrombosis rates of 0.5% in emergencies [51].

(B) *Selective Shunts*. Selective shunts aim to decompress only the gastrosplenic circulation leaving the blood flow to the liver intact, therefore, theoretically at least decreasing the rebleeding risk from oesophagogastric varices whilst preserving hepatopetal flow. The distal splenorenal shunt (Warren shunt) is the most commonly performed selective shunt although other shunts also have been described like the left gastroepiploic to left renal vein and left gastric to left renal vein shunts. Warren et al. reported a 5 yr followup of patients treated with distal splenorenal shunts. The patency rates were 92%, rebleeding rates of 12%, shunt dysfunction occurred in 25%, and encephalopathy in none of their patients [12]. These good results have been validated by others [52].

The main disadvantages are the need for surgical expertise and increased operation time. Such shunts cannot be performed in patients with thrombosis of the splenic vein or those who have a history of splenectomy. 

*Rex Shunt*. Superina et al. in a study of 34 patients showed that mesenterico left portal vein bypass (MLPVB) or Rex shunt was successful in 91%, and they concluded that it was a more physiological shunt for EHPVO [13]. They claim that the shunt results in liver growth and normalization of coagulation parameters. But it requires the presence of a patent superior mesenteric vein, intrahepatic left portal vein, and internal jugular vein. However, it is still not popular in developing countries.

#### 4.3.2. Variceal Ablative Procedures

 These include splenectomy either alone or in combination with oesophagogastric devascularisation. These procedures are indicated if (i) performed as salvage therapy in variceal bleeding not controlled with endoscopic measures, (ii) a suitable size vein is not available for a shunt procedure, (iii) surgical expertise for a shunt procedure is not available. Splenectomy alone is not recommended since it does not decompress the portal circulation and also leads to thrombosis of splenic vein which thereafter cannot be used for shunting later.

Mathur et al. evaluated the role of oesophagogastric devascularisation with or without gastro-oesophageal stapling in acute variceal bleeding [34]. In their study, 20 patients had EHPVO and the operative mortality was 5%; recurrent varices occurred in 5% with rebleeding in 11%. None of their patients had encephalopathy. In a retrospective study of 24 patients with EHPVO undergoing salvage surgery for variceal bleeding (13 had devascularisation procedures; 11 had proximal splenorenal shunts), they achieved control of bleeding in 96%. One patient who had continued to bleed after surgery died [35]. Goyal et al. retrospectively reported a four-year followup of 22 patients with noncirrhotic portal hypertension in whom the bleeding was not controlled with endoscopic therapy. In their study, the rebleeding rate was 10% and overall survival was 95% [36]. 

*Summary*. EHPVO more commonly involves children from the lower socioeconomic strata in developing countries. Variceal bleeding is the most common presentation. Endoscopy is the established treatment for acute control of bleeding. Salvage surgery after failure of endoscopy has also shown good results. Both endoscopy and shunt surgeries have shown good long-term results in secondary prophylaxis. However, treatment should take into account the socioeconomic status of the patient and facilities available locally. Splenectomy and proximal lienorenal shunt being a one-time procedure with, if performed by experienced surgeons, low mortality and occlusion rates and an absence of postprocedure encephalopathy should be considered as the main treatment in patients with difficult access to sophisticated medical facilities. The role of shunt surgery has expanded since it is also effective in correcting portal biliopathy, hypersplenism, and growth retardation and may improve the quality of life.

## Figures and Tables

**Table 1:** Followup results with endoscopic variceal eradication therapy.

Author (yr)	No. of patients	Eradication (%)	Rebleed (%)	Recurrence (%)	Followup period
Howard et al. (1988) [3]	33	100	9	6	35 months
Chawla et al. (1990) [4]	122	91	16	6	24 months
Stringer and Howard (1994) [5]	32	100	31	16	8.7 years
Zargar et al. (2004) [6]	69	91	12	14	15 years
Thomas et al. (2009) [7]	198	100	17	19	20 years

**Table 2:** Outcomes of different shunt surgeries in EHPVO.

Author (yr)	Type of shunt performed	No. of patients	Shunt patency (%)	Rebleed (%)	Encephalopathy (%)	Operative mortality (%)	Follow up period	Survival
Mitra et al. (1993) [8]	Side-side lienorenal shunt (SSLR)	81	84	11	Nil	Nil	54 months	100%
Prasad et al. (1994) [9]	Proximal splenorenal shunt (PSRS)	160	NA	11	Nil	1.9	Up to 15 yrs	95% at 15 yrs
Orloff et al. (1994) [10]	PSRS, SSLR, and mesocaval shunt	162	98	2	Nil	Nil	Up to 35 yrs	96% at 10 yr
Rao et al. (2004) [11]	SSLR, PSRS	20	95	Nil	Nil	NA	3–5 yrs	95%
Warren et al. (1988) [12]	Distal splenorenal shunt	25	96	12	Nil	Nil	60 months	96%
Superina et al. (2006) [13]	Rex shunt	34	95	NA	Nil	NA	Up to 7 yrs	100%
Sharif et al. (2010) [14]	Rex shunt	24	96	2	NA	Nil	5.3–8.8 yrs	NA

NA: not available.
